# Prediction of Mental Health Problem Using Annual Student Health Survey: Machine Learning Approach

**DOI:** 10.2196/42420

**Published:** 2023-05-10

**Authors:** Ayako Baba, Kyosuke Bunji

**Affiliations:** 1 Health Service Center Kanazawa University Ishikawa Japan; 2 Graduate School of Business Administration Kobe University Hyogo Japan

**Keywords:** student counseling, health survey, machine learning, mental health problem, response time

## Abstract

**Background:**

One of the reasons why students go to counseling is being called on based on self-reported health survey results. However, there is no concordant standard for such calls.

**Objective:**

This study aims to develop a machine learning (ML) model to predict students’ mental health problems in 1 year and the following year using the health survey’s content and answering time (response time, response time stamp, and answer date).

**Methods:**

Data were obtained from the responses of 3561 (62.58%) of 5690 undergraduate students from University A in Japan (a national university) who completed the health survey in 2020 and 2021. We performed 2 analyses; in analysis 1, a mental health problem in 2020 was predicted from demographics, answers for the health survey, and answering time in the same year, and in analysis 2, a mental health problem in 2021 was predicted from the same input variables as in analysis 1. We compared the results from different ML models, such as logistic regression, elastic net, random forest, XGBoost, and LightGBM. The results with and without *answering time* conditions were compared using the adopted model.

**Results:**

On the basis of the comparison of the models, we adopted the LightGBM model. In this model, both analyses and conditions achieved adequate performance (eg, Matthews correlation coefficient [MCC] of with *answering time* condition in analysis 1 was 0.970 and MCC of without *answering time* condition in analysis 1 was 0.976; MCC of with *answering time* condition in analysis 2 was 0.986 and that of without *answering time* condition in analysis 2 was 0.971). In both analyses and in both conditions, the response to the questions about campus life (eg, anxiety and future) had the highest impact (Gain 0.131-0.216; Shapley additive explanations 0.018-0.028). Shapley additive explanations of 5 to 6 input variables from questions about campus life were included in the top 10. In contrast to our expectation, the inclusion of answering time–related variables did not exhibit substantial improvement in the prediction of students’ mental health problems. However, certain variables generated based on the answering time are apparently helpful in improving the prediction and affecting the prediction probability.

**Conclusions:**

These results demonstrate the possibility of predicting mental health across years using health survey data. Demographic and behavioral data, including answering time, were effective as well as self-rating items. This model demonstrates the possibility of synergistically using the characteristics of health surveys and advantages of ML. These findings can improve health survey items and calling criteria.

## Introduction

### Background

In Japan, 57% of female and 48% of male students seek counseling voluntarily [1]. Others seek counseling after being referred by teachers, families, friends, or health service centers. Health service centers use health surveys as clues for counseling calls. A health survey is mainly conducted as a self-reported screening test or an interview along with health checkups [2].

The style of mental health surveys differs among universities. Participants in the survey range from first-year students to undergraduate students to all students, including graduate students. The content is also diverse, with the University Personality Inventory (UPI) [3] and General Health Questionnaire (GHQ) [4,5] being frequently used [6]. Similarly, the criteria for calling using a mental health survey are inconsistent [7]. It is challenging to devise uniform criteria that will aid in efficient calling. To address this issue, we propose that machine learning (ML) would aid in developing a universal method for detecting and predicting mental health problems among students.

ML has several advantages in modeling highly individual phenomena with many variables, such as allowing the simultaneous testing of numerous input variables and their complex interactions [8], permitting nonlinearity in producing predictive algorithms [9], and having the ability to test all possible relationships to identify the superlative algorithm and model without a priori hypothesis by researchers [8]. These advantages of ML can help detect and predict mental health problems.

### Related Work

Many trials detect mental health problems using biological, behavioral, and subjective data [10,11]. Some studies have diagnosed suicidal ideation or behavior using various predictors, such as psychological problems and self-injuries [12,13]. To predict mental health problems, the following broader variables were tested: birth information; physical illness; environmental factors; behavioral data (eg, number of naps, study duration, and use of a cell phone); biological data (eg, sleep onset time, skin conductance, and temperature) collected with wearable sensors, and text data posted on social media [14-17].

There are also some examples of ML with on-campus data, known as learning analytics and educational data mining [18]. In some studies, repeaters and dropouts have been predicted using log data from a learning management system or e-learning, information about entrance examinations, registered courses, attendance, grades, and submission status of assignments [19-24].

On-campus mental health surveys are insufficiently used as ML predictors. We consider them suitable for statistical analysis for 3 reasons. First, the mental health survey is open to all students, and the response rate is high. They must complete it to receive health checkups. Second, mental health surveys are conducted annually at many universities. Thus, it is possible to observe a change over time. Third, web-based mental health surveys, which have become a standard under COVID-19, can collect well-organized self-reported data and *answering time* data.

We use the word *answering time* as a broad concept, including response time (RT), which is the amount of time in which a student responds to the question item after it is shown on the screen; RT stamp (the time of day when a student accesses the survey form); and answer date (the number of days elapsed in which a student completes the survey after it was announced). In particular, RT has recently attracted attention mainly in psychological assessment and educational testing; however, little is known about how to use RT in mental health measurement [25,26]. A few studies have shown that RT is related to depression estimation and the prediction of suicide ideation and attempt status [25,27]. RT would be worth investigating for predicting mental health based on the response behavior.

### Goal of This Study

This study aimed to predict students’ mental health problems even without a specific mental health score. In this study, we conducted 2 analyses. In analysis 1, we attempted to predict mental health problems from other items when responding to the survey. Analysis 2 attempted to predict mental health problems in 2021 from the responses in the survey conducted in 2020 (1 year later). We also tested the effect of answering time on the prediction of mental health problems.

The main objective of this study was to build an exploratory predictive model. We also attempted to evaluate the impact of each input variable on the outcome based on ML and examine the interpretable relationship between input variables and mental health problems.

This study demonstrates the possibility of using data collected from health surveys conducted at most universities to precisely detect students experiencing mental health problems and reduce call costs. This will also pave the way to using existing health survey data even without mental health scores.

## Methods

### Data Collection

The data set was obtained from a closed health survey conducted at the beginning of 2020 and 2021 school years at University A, a national university in Japan. According to the email announcement of the annual health checkup, all students were asked to answer the survey on the learning management system. Although incentives were not offered and penalties were not levied, the students were required to answer this survey to obtain a health certification. However, the survey did not comprise adaptive questions. There were 28 nonrandomized questions (health survey in Table 1) that were presented one per page without completeness check. Students could review their answers before submission and resubmit them before the deadline. When there were duplicate submissions from identical IDs, the last submission was recorded.

**Table 1:** List of items in the health survey.

Item				Options
**Individual data**				
	Faculty and department			24 categories
	Sex			Male and female
	International student			Yes and no
	Age			(Free description)
	Years of university			1, 2, 3, 4, 5, and 6
**Health survey**				
	**Case history**			Yes, no, and do not know
		Circulatory problem		
		Digestive problem		
		Cranial nerve and mental disorders		
		Respiratory problem		
		Allergic disease		
		Measles		
		Rubella		
		Mumps		
		Varicella		
		Other diseases		
	Currently under medical treatment			Yes and no
	Health concerns or worries			Yes and no
	Physical or mental disability			Yes and no
	Physical or mental disability certificate			Yes and no
	Needs for consultation or support from the university			Yes, no, and do not know
	Blood type			A, B, O, AB, and do not know
	Tuberculin skin test result			Negative, positive, and do not know
	**Vaccination**			1 time, ≥2 times, never, and do not know
		Measles		
		Rubella		
		Mumps		
		Varicella		
	**Have meals**			Every day, sometimes, and never
		Breakfast		
		Lunch		
		Dinner		
	Sleeping hours			15 categories (1-hour increments; from <3 to >17 hours)
	Exercise			Every day, sometimes, and never
	Drinking			Every day, sometimes, and never
	Smoking			Yes and no
	Thoughts on quitting smoking			Want, do not want, and nonsmoker
	Residence			Home, apartment, student dormitory, and others
	Commuting method			Walking, bus, train and bus, bicycle, motorbike, car, and others
	Club activities			Yes, used to be, and never
	Hours of internet use			20 categories (1-hour increments; from <0.5 to >19 hours)
	**Nine questions about campus life**			Yes and no
		This is not the university or major that I wanted.		
		I have a strong anxiety about my campus life.		
		It takes me a long time to get used to new surroundings.		
		I am worried about my relationships with other people.		
		I am worried about my future.		
		I had some trouble with someone close to me.		
		I feel like my life rhythm has been upset recently.		
		I am worried that I may not be able to take credit as I think.		
		I always feel busy with my academic work.		
	Kessler 6 (mental health scale) [28]			4=all of the time, 3=most of the time, 2=some of the time, 1=a little of the time, and 0=none of the time
	**Have lost >3 kg in a month**			Yes and no
		Presently		
		In the past (>18 years old)		
		In the past (<18 years old)		
	What you want to tell us and questions			Free description
	Menstrual trouble (for women only)			Yes and no
	Coping with menstrual trouble (for women only)			Seeing a physician at a hospital, want to consult Health Service Center, and would like to wait and see how it goes
	Menstrual cycle (for women only)			Regular every 26-32 days, not every 26-32 days but comes regularly, not regular but more than once every 3 months, and not more than once every 3 months
	Menstrual pain (for women only)			No pain, no need to take painkillers, need to take painkillers, and painkillers do not alleviate my pain
	**Birth-control pills or oral contraceptives (for women only)**			Yes and no
		Taking		
		To improve menstrual cycle		
		To improve menstrual pain		
		For other reasons		

In this study, data that met the following conditions were excluded: during the 2020 or 2021 survey, the participant was, not an undergraduate, did not answer by the deadline (27 days in 2020 and 39 days in 2021), did not respond to any items, did not respond to all items on the Kessler 6 mental health scale [29] (it was used as an outcome and detailed in Outcome and Input Variables section), was a nonregular student (eg, credited auditors), and did not give consent for data use. Incomplete submissions or atypical response duration were not excluded.

In 2020 and 2021, a total of 5690 students may have been affiliated with the university based on student enrollment conditions in 2020. In both the 2020 and 2021 surveys, 3680 students answered the survey. In total, 119 (3.23%) students’ data were excluded: 10 (8.4%) students did not consent, 105 (88.2%) students did not complete Kessler 6 scale in 2020 or 2021 survey, and 4 (3.3%) students belonged to old affiliations. The resulting data set used for the analysis contains 3561 students (Table 2).

The mean age was 20.54 (SD 1.75) years. The distribution of students by sex was 1941 (54.51%) male and 1620 (45.49%) female students. The data set included 1234 (34.65%) first-year students, 1005 (28.22%) second-year students, 1018 (28.59%) third-year students, and 304 (8.54%) fourth- or higher-year students. The distribution of students by faculty was 1361 (38.22%) in humanities and social sciences (6 departments); 943 (26.48%) in science and engineering (8 departments); 1175 (33%) in medical, pharmaceutical, and health (4 departments); and 82 (2.3%) in others (2 departments). Furthermore, 10 (0.28%) students were international students.

**Table 2:** Demographic characteristics of participants in the 2020 school year (N=3561).

Variables		Values
Age (years), mean (SD)		20.54 (1.75)
**Sex, n (%)**		
	Male	1941 (54.51)
	Female	1620 (45.49)
**Years of university, n (%)**		
	First-year students	1234 (34.65)
	Second-year students	1005 (28.22)
	Third-year students	1018 (28.59)
	Fourth-year or higher-year students	304 (8.54)
**Faculty, n (%)**		
	Humanities and social sciences (6 departments)	1361 (38.22)
	Science and engineering (8 departments)	943 (26.48)
	Medical, pharmaceutical, and health (4 departments)	1175 (33)
	Others (2 departments)	82 (2.3)
International students, n (%)		10 (0.28)

### Ethics Approval and Informed Consent

This study received ethics approval from the Medical Ethics Committee of Kanazawa University (test number:2011-113 [017]). All students were asked to complete the survey; however, they had the option to not consent for its use for research purposes. We obtained written informed consent from all participants. Data were anonymized and saved offline. The usability and technical functionality were tested by staff members in advance. The research was performed according to relevant guidelines and regulations.

### Outcome and Input Variables

Our model did not use the diagnosis but used the Japanese version of the Kessler 6 [28] as an indicator of mental health problems. Diagnoses of the students were not necessarily shared with the university, whereas Kessler 6 could be obtained from the health survey conducted in the university. The Japanese version of the Kessler 6 demonstrates high performance in detecting mood or anxiety disorders according to the Diagnostic and Statistical Manual of Mental Disorders Fourth Edition [28,30]. Although diagnoses are not made based only on Kessler 6 scores and need consultations with physicians, they have been used widely for screening and surveys [31]. Analyses 1 and 2 used the Kessler 6 scores for 2020 and 2021, respectively, as outcomes. Both analyses divided scores into a dichotomous variable (1: problem and 0: no problem) according to a ≥13 cutoff point [29,30].

In this study, we examined several candidate ML models to determine which model to use. All candidate models are probabilistic prediction models: logistic regression, elastic net, random forest, XGBoost, and LightGBM. Elastic net is a logistic regression model with L1 and L2 regularization. Random forest combines independent decision trees and outputs the most popular class as a prediction [32]. XGBoost and LightGBM are implementations of the gradient boosting decision tree (GBDT) [33,34]. The GBDT is known to achieve high performance in many ML tasks, such as predicting diseases [35] or identifying psychological health risk factors [36].

We used the same input variables for analyses 1 and 2. All the responses to the 2020 questionnaire listed in Table 1 were coded differently according to the model. For tree-based models (random forest, XGBoost, and LightGBM), they were coded numerically, with missing values filled with −99. For example, the answer for *blood type* item, which was one among A, B, O, AB, do not know, and no response, was coded as 1, 2, 3, 4, 5, and −99, respectively. For the regression models (logistic regression and elastic net), different coding schemes were applied based on the type of input variables. Continuous or ordinal variables such as *hours of internet use* or *age* were numerically coded in the same manner as tree-based models, except that missing or nonresponse data were filled with the grand mean. In contrast, effect coding was applied to nominal variables such as *case history* or *blood type*. Missing data were treated as independent categories. As a result, the response to *blood type* (Q7), for instance, was transformed into 5 input variables: ANS7_B, ANS7_O, ANS7_AB, ANS7_DN (do not know), and ANS7_NA (missing). When the respondent answered “B,” ANS7_B was coded as 1 and the remaining 4 were coded as 0.

Because the aim of this study was to investigate which type of input variables were useful in the prediction of mental health problems, we used all variables except RT without dimensionality reduction. Notably, the tree-based models are less susceptible to the inclusion of irrelevant variables. In addition, an elastic net can reduce the number of input variables because of regularization.

In addition to the raw response variables in Table 1, we generated several input variables with the expectation that some patterns would appear on each variable, as shown in Table 3. These conjectures are based on existing studies on students’ mental health and suicide [37], shared knowledge among experts, or authors’ clinical experiences at the university.

To deal with the content of answers to the free description question (*What you want to tell us and questions*), the answers were analyzed using KH Coder [38,39], a free text mining analysis software. First, words that appeared ≥10 times were clustered using the Ward method, and Jaccard distances between clusters were calculated. Six clusters were adopted for interpretation. Second, each free description was labeled as 0 or 1 for each cluster. If words in a cluster did not appear in a free description, the description was labeled as 0 for the cluster; if words contained in the cluster appeared even once in a free description, the description was labeled as 1 for the cluster. Cluster 1 contained, among other words, “counseling,” “current,” and “under treatment.” Cluster 2 contained, among other words, “syndrome,” “surgery,” and “diagnosis.” Cluster 3 contained, among other words, “menstruation” and “anemia.” Cluster 4 contained, among other words, “left,” “ear,” and “right.” Cluster 5 contained, among other words, “depression,” “mental,” “stress,” and “anxiety.” Cluster 6 contained, among other words, “hospital,” “allergy,” and “skin.”

This study examined the effectiveness of answering time in predicting mental health problems. Table 4 lists the answering time–related input variables.

The 2 coordinates Xs and Ys represent the position of the time stamp in a 24-hour clock and are introduced to indicate that 0:00 and 24:00 are identical. They were calculated by


X_s_ = cos([s / 43200]π), Y_s_ = sin([s / 43200]π) **(1)**

where *s* is the number of seconds elapsed in a day; for instance, when the time stamp is 13:34:42, *s* will be 13 × 3600 + 34 × 60 + 42 = 48882. RT-related variables were included as the first and second principal component scores because the RT of every single item is difficult to interpret, and these RTs can moderately affect the prediction.

Except for the text-mining analysis, the abovementioned feature engineering processes were executed using the R programming language [40] on a Windows 10 machine, with the missMDA package [41] for principal components analysis with missing data.

**Table 3:** Generated variables and possible conjecture.

Item	Conjecture
The academic year of admission (the values in the data were between 2012 and 2020)	There may be a cohort effect.
An indicator to show how many years after age 18 (standard in Japan) the student was enrolled (2750 students were never retained , while 811 were retained)	Some enrollments over the standard age may have reflected or caused some problems including mental health problems.
An indicator to show how many years the respondent had ever been retained (3527 students were never retained, while 34 were retained)	Some students who repeated school years may have had some mental health problems.
The number of unanswered items	Students with mental health problems may hesitate to answer or miss many items.
The number of characters in answer to the free description question, *what you want to tell us and questions*	Students with mental health problems may write a lot in free descriptions to make the university aware of their difficulty.
Dummy variables for including 6 specific word clusters^a^	Some students with mental health problems may have used specific words in common and expressions in the free description.
Number of “yes” answers in *case history* and *nine questions about campus life*	Students with mental health problems may have experienced some diseases or campus life problems.
The proportion of “yes” answers to each item in *nine questions about campus life* in the respondent’s department	Some students who belonged to departments with many students with campus life problems may have felt stress.
The number of students and the proportion of same-sex students in the respondent’s department	Some students who belonged to the departments which have few same-sex students may have had difficulty in communication and relationships.

^a^Cluster 1 contained, among other words, “counseling,” “current,” and “under treatment.” Cluster 2 contained, among other words, “syndrome,” “surgery,” and “diagnosis.” Cluster 3 contained, among other words, “menstruation” and “anemia.” Cluster 4 contained, among other words, “left,” “ear,” and “right.” Cluster 5 contained, among other words, “depression,” “mental,” “stress,” and “anxiety.” Cluster 6 contained, among other words, “hospital,” “allergy,” and “skin.”

**Table 4:** Generated variables based on answering time and possible conjecture.

Item	Conjecture
Answer date (the number of days elapsed between the survey announcement and the completion of survey by a student)	Students with mental health problems (eg, procrastination traits and, lack of information gathering) may answer the survey later.
RT^a^ per character in the free description question *what you want to tell us and questions*	Some students who wrote smooth free descriptions may have had some health problem to tell the university.
2D-dimensional coordinates of response time stamps^b^	Response time stamps may have reflected students’ life rhythm and sleep quality, which may be related to mental health state.
Kernel density of *s*	Some students who answered at different times compared with others may have had problems with life rhythm and sleep quality, which may be related to mental health problems.
2 principal components scores of log-transformed standardized RTs of all items	Students with mental health problems may spend a longer or shorter time on specific items than other students.
Individual mean and SD of standardized log RTs calculated using all valid responses	Students with mental health problems may spend, on average, a longer or shorter time answering. Students with mental health problems may spend very different time on different items.
2 principal components scores of differences between the standardized log RTs of all items and the individual mean	Students with mental health problems may spend longer or shorter time on answering specific items than others.
2 principal components scores of kernel density of the standardized log RTs of all items	Students with mental health problems may show peculiar RT patterns.

^a^RT: response time.

^b^They were calculated with equation 1.

### Procedure

In this procedure, we did not split the data into training and test data. This is because the values for the obtained metrics varied considerably depending on the data split owing to data imbalance. Thus, only 5.5% (196/3561) of the students in 2020 and 6.88% (245/3561) in 2021 had a higher risk of mental illness problems as assessed by Kessler 6, including 2.33% (83/3561) of the students who had problems both in 2020 and 2021. Typically, such imbalanced data are dealt with by applying sample weight to equal the total number of positive and negative observations. However, our preliminary comparison performed better when we did not impose sample weights on all candidate models. Therefore, we did not use any particular procedures for dealing with imbalanced data.

**Figure 1:**
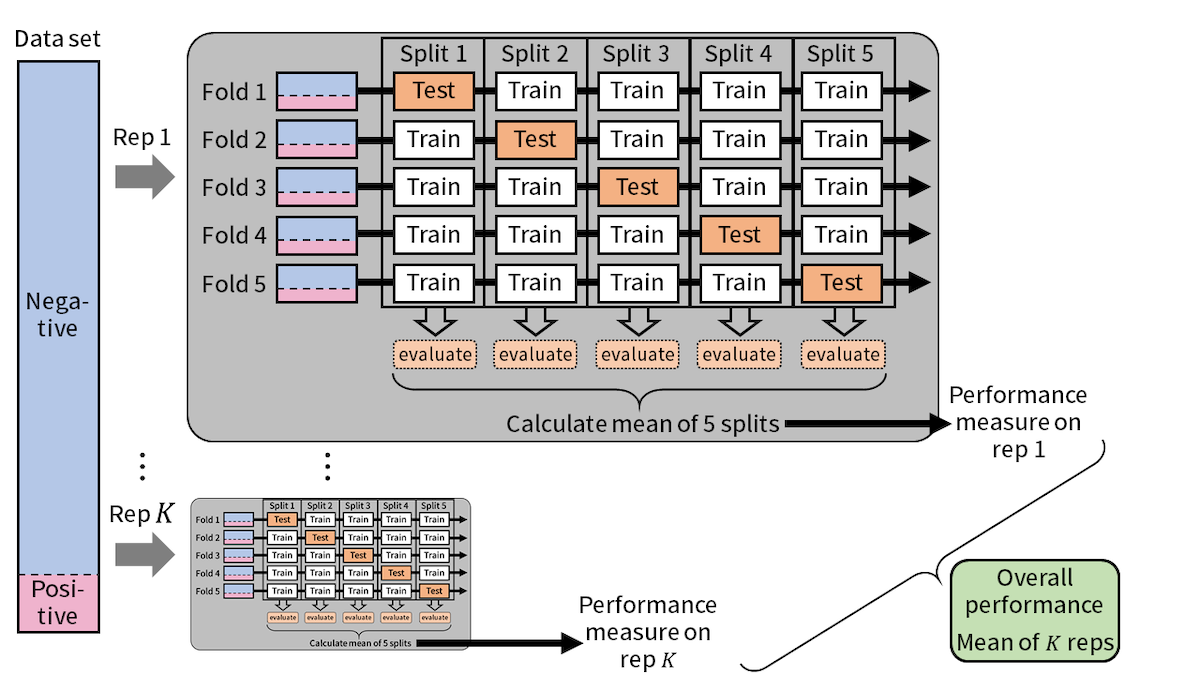
Flowchart of 5-fold cross-validation in each part.

First, we compared the performance of candidate ML models to determine which model to use. The procedure was divided into 2 parts: parameter tuning and performance evaluation. Both parts were conducted based on the following procedure (*K* repeats of 5-fold cross-validation; Figure 1 shows the schema of the procedure):

1. Randomly shuffle the entire data set and split it into 5 folds so that the positive:negative ratio of the outcome variable is nearly the same among the data sets.
2. Choose 1 of the 5 data sets as the test data, and train the model using the remaining 4 data sets.
3. Evaluate the trained model on the test data.
4. Repeat steps 2 and 3 for each data set.
5. Calculate the means of 5 data sets on each evaluation metric.
6. Repeat steps 1 through 5 *K* times.
7. Calculate the means of *K* repeats on each evaluation metric.

In the parameter tuning part, the model’s hyperparameters were optimized based on the tree-structured Parzen estimator [42]. The log-loss, as a model performance measure, in a specific parameter set was measured by *K*=50 repeats of 5-fold cross-validation. Log-loss at fold *f* of the *k*-th repeat is calculated by


LogLoss(k,f) = − (1 / n)Σ([y_i_]log[p_i_] + [1 − y_i_]log[1 − p_i_]) **(2)**

where *y_i_* and *p_i_* are the realized value and predicted probability of respondent *i*, respectively, and *n* is the number of observations in the fold *f*. As a result, the log-loss for a specific parameter set was obtained by


LogLoss = (1 / K)Σ(1 / 5)ΣLogLoss(k,f) **(3)**

All the hyperparameters tuned in this study are summarized in Table 5 [43-46]. These parameters can cope with overfitting if they are appropriately tuned. In addition, the learning rate parameters in the GBDT models (eta in XGBoost and learning rate in LightGBM) were fixed at 0.01. Generally, the smaller the learning rate, the more accurate is the prediction, although the computation time increases.

As a result, the performance in each parameter set was measured as the average of *K*=50 log-losses. Finally, the parameter values that minimize the objective performance measure were adopted, and we proceeded to the performance evaluation. By using the optimized parameter set, we obtained 8 model performance measures (log-loss, Brier loss, area under the curve of receiver operating characteristic curve [AUC-ROC], area under the curve of precision-recall curve [AUC-PR], specificity, precision, recall [also known as sensitivity], and Matthews correlation coefficient [MCC]). Log-loss, Brier loss, AUC-ROC, and AUC-PR are measures based on predictive probability (*p_i_*). In contrast, the specificity, precision, recall, and MCC are obtained from the confusion matrix. For example, MCC is calculated as follows:

*(TP* × *TN* – *FP* × *FN) / √(*[*TP + FP*] × [*TP + FN*] × [*TN + FP*] × [*TN + FN*]*)* **(4)**

where *TP, TN, FP,* and *FN* are the numbers of true-positive, true-negative, false-positive, and false-negative respondents, respectively. As previously mentioned, this study used imbalanced data. Therefore, we included the AUC-PR and MCC, which are commonly used in such cases [47].

On the basis of the performance measures obtained, we selected the best model. To test the extent to which answering time–related variables contribute to prediction performance in the selected model, we also conducted parameter tuning and performance evaluation phases without all answering time–related variables (shown in Table 4). We call the analysis without answering time–related variables “*without* condition, whereas the analysis with all input variables is called *with* condition.

Furthermore, we calculated feature importance measures (Gain and Shapley additive explanations [SHAP]) on both *with* and *without* conditions. SHAP is based on the Shapley values, the average marginal contribution in game theory. With the firm theoretical basis of game theory, SHAP can explain the extent to which each input variable contributes to the model’s prediction [48]. Although several approaches exist for interpreting the prediction, we used SHAP because it is easy to implement and understand. Using the SHAP dependence plot, we can understand the relationship between the actual input value and its effect on the predicted probability. The abovementioned procedures were executed using all the data in *K*=100 repeats of 5-fold cross-validation. After model evaluation, we obtained SHAP dependence plots on several variables to interpret the model prediction.

All the procedures described in this section were executed using the Julia programming language [49] with several libraries: MLJ.jl [50], TreeParzen.jl [51], ScikitLearn.jl [52], LightGBM.jl [53], and ShapML.jl [54]. Compared with several famous languages, such as R and Python, Julia is still a young language and is rapidly developing. However, it is already perceived as sufficiently stable to be used for research purposes [55,56].

**Table 5:** Hyperparameters of each model tuned in the parameter tuning part.

Model and hyperparameter			Description
**Elastic net**			
	gamma	Strength of the L1 regularization	
	lambda	Strength of the L2 regularization	
**Random forest**			
	n_estimators	The number of trees in the forest	
	max_depth	The maximum depth of the tree	
	min_samples_leaf	The minimum number of samples required to be at a leaf node.	
	max_features	The proportion of features used in each tree	
	max_samples	The proportion of samples used in each tree	
**XGBoost**			
	num_round	The number of boosting iterations	
	max_depth	The maximum depth of the tree	
	min_child_weight	Minimum sum of instance weight needed in a child	
	alpha	Strength of the L1 regularization	
	lambda	Strength of the L2 regularization	
	colsample_bytree	The proportion of features used in each tree	
	subsample	The proportion of samples used in each tree	
**LightGBM**			
	num_iterations	The number of boosting iterations	
	num_leaves	The maximum number of leaves in 1 tree	
	max_depth	The maximum depth of the tree	
	min_deta_in_leaf	The minimal amount of data in 1 leaf	
	lambda_l1	Strength of the L1 regularization	
	lambda_l2	Strength of the L2 regularization	
	feature_fraction	The proportion of features used in each tree	
	bagging_fraction	The proportion of samples used in each tree	

## Results

**Figure 2:**
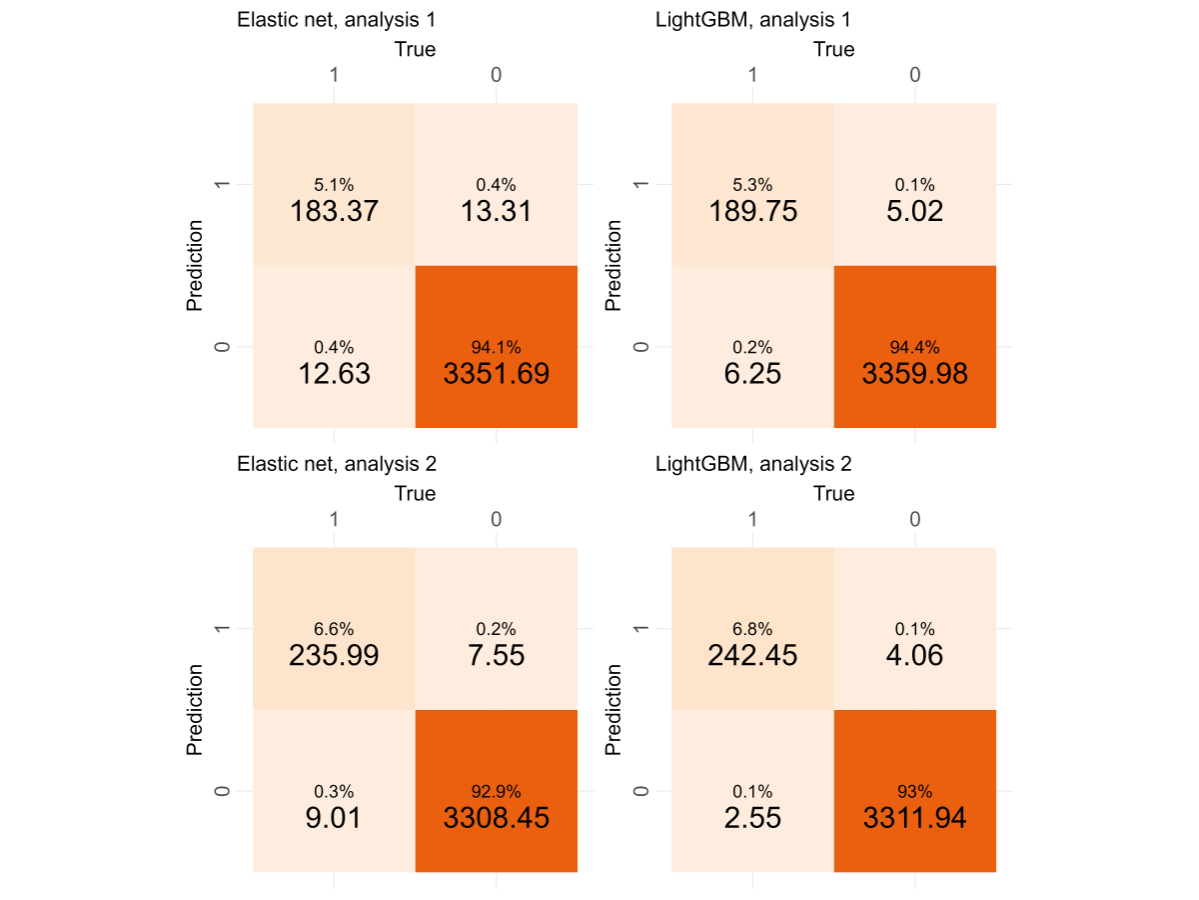
Confusion matrices on the elastic net (left half) and LightGBM (right half).

Tables 6 and 7 summarize mean and SD of each model’s performance measures. As explained in *Procedure* section, the resultant values shown in Tables 6 and 7 are the means and SDs calculated from 100 trials. Italicized variables indicate the best model in terms of each measure. Evidently, the elastic net and LightGBM performed best. Regarding probabilistic measures (Table 6), elastic net afforded slightly better values than LightGBM. By contrast, binary measures, which were calculated from the confusion matrix (Table 7), were apparently better at LightGBM. Figure 2 shows the confusion matrix obtained from the elastic net and LightGBM in analyses 1 and 2. The actual values given below the percentages in each cell are the averages of K=100 repeats. In analysis 1, LightGBM misclassified only 11.27 (SD 1.75) observations (5.02, SD 1.58 false positives and 6.25, SD 1.18 false negatives) out of 3561 on average, whereas elastic net misclassified more than twice (average 25.94, SD 2.49) as many observations as LightGBM. Similar results were obtained in analysis 2. On the basis of these outcomes, we report the results for LightGBM.

Table 8 presents a comparison of the performances of LightGBM measures between *with* and *without* conditions. In contrast to our expectations, these results were mixed. This implies that the use of answering time–related variables does not necessarily improve the prediction.

Tables 9-12 show the top 10 input variables ranked by Gain and SHAP importance. SHAP importance was calculated as the average absolute value of all respondents. The number of “yes” answers to nine questions about campus life had the highest impact regardless of the analyses, conditions, and feature importance measures (Tables 9-12). In Table 9 (Gain in with condition), 5 of the top 10 input variables are answering time–related variables. In Table 10 (Gain in without condition), hours of internet use, years of university, and proportion of the same sex in the department were ranked common to both analyses 1 and 2, in addition to variables related to nine questions about campus life. In Table 11 (SHAP in with condition), input variables from nine questions about campus life and answering time–related input variables mainly occupied the top 10 variables in analysis 1. In analysis 2, answering time–related input variables were out of rank, whereas years of university, the academic year of admission, diet-related variables, and proportion of the same sex in the department were ranked. In Table 12 (SHAP in without condition), years of university, academic year of admission, and diet-related variables were ranked common to both analyses 1 and 2 in addition to variables related to nine questions about campus life.

**Table 6:** Probabilistic performance measures of each model.

		Log-loss, mean (SD)	Brier loss, mean (SD)	AUC-ROC^a^, mean (SD)	AUC-PR^b^, mean (SD)
**Analysis 1**					
	Logistic regression	0.252 (0.012)	0.109 (0.003)	0.760 (0.012)	0.215 (0.013)
	Elastic net	*0.164 (0.001)* ^c^	*0.090 (0.001)*	*0.862 (0.003)*	*0.292 (0.010)*
	Random forest	0.210 (0.008)	0.113 (0.001)	0.833 (0.005)	0.218 (0.010)
	XGBoost	0.165 (0.001)	0.091 (0.001)	0.855 (0.003)	0.278 (0.009)
	LightGBM	0.165 (0.001)	*0.090 (0.001)*	0.857 (0.003)	0.281 (0.010)
**Analysis 2**					
	Logistic regression	0.342 (0.015)	0.140 (0.003)	0.696 (0.009)	0.178 (0.011)
	Elastic net	*0.211 (0.001)*	*0.115 (0.001)*	*0.796 (0.004)*	*0.264 (0.008)*
	Random forest	0.250 (0.002)	0.140 (0.001)	0.768 (0.005)	0.200 (0.007)
	XGBoost	0.213 (0.001)	0.116 (0.001)	0.791 (0.004)	0.249 (0.008)
	LightGBM	0.213 (0.001)	0.116 (0.001)	0.789 (0.004)	0.246 (0.009)

^a^AUC-ROC: area under the curve of receiver operating characteristic curve.

^b^AUC-PR: area under the curve of precision-recall curve.

^c^Italicized variables indicate the best model in terms of each measure.

**Table 7:** Performance measures on each model from confusion matrix.

		Specificity, mean (SD)	Precision, mean (SD)	Recall, mean (SD)	MCC^a^, mean (SD)
**Analysis 1**					
	Logistic regression	0.977 (0.002)	0.681 (0.020)	0.823 (0.015)	0.732 (0.016)
	Elastic net	0.996 (0.001)	0.934 (0.009)	0.936 (0.009)	0.931 (0.007)
	Random forest	0.976 (0.002)	0.681 (0.022)	0.859 (0.018)	0.749 (0.017)
	XGBoost	0.998 (0.001)	0.963 (0.008)	0.963 (0.006)	0.961 (0.005)
	LightGBM	*0.999 (0.000)* ^b^	*0.975 (0.008)*	*0.968 (0.006)*	*0.970 (0.005)*
**Analysis 2**					
	Logistic regression	0.976 (0.002)	0.730 (0.018)	0.884 (0.012)	0.787 (0.013)
	Elastic net	0.998 (0.001)	0.970 (0.006)	0.963 (0.005)	0.964 (0.004)
	Random forest	0.967 (0.002)	0.662 (0.017)	0.857 (0.015)	0.732 (0.014)
	XGBoost	0.998 (0.000)	0.977 (0.005)	0.987 (0.004)	0.981 (0.004)
	LightGBM	*0.999 (0.000)*	*0.984 (0.005)*	*0.990 (0.004)*	*0.986 (0.003)*

^a^MCC: Matthews correlation coefficient.

^b^Italicized variables indicate the best model in terms of each measure.

**Table 8:** Performance measures of with and without conditions on LightGBM.

Measure	Analysis 1			Analysis 2	
	*With*, mean (SD)	*Without*, mean (SD)	*With*, mean (SD)		*Without*, mean (SD)
Log-loss	*0.165 (0.001)* ^a^	*0.165 (0.001)*	0.213 (0.001)		*0.211 (0.001)*
Brier loss	*0.090 (0.001)*	*0.090 (0.001)*	0.116 (0.001)		*0.115 (0.001)*
AUC-ROC^b^	0.857 (0.003)	*0.858 (0.003)*	0.789 (0.004)		*0.798 (0.004)*
AUC-PR^c^	*0.281 (0.010)*	0.276 (0.010)	0.246 (0.009)		*0.260 (0.010)*
Specificity	*0.999 (0.000)*	0.998 (0.000)	*0.999 (0.000)*		0.998 (0.001)
Precision	*0.975 (0.008)*	0.974 (0.008)	*0.984 (0.005)*		0.970 (0.007)
Recall	0.968 (0.006)	*0.981 (0.007)*	*0.990 (0.004)*		0.977 (0.005)
MCC^d^	0.970 (0.005)	*0.976 (0.005)*	*0.986 (0.003)*		0.971 (0.004)

^a^Italicized variables indicate the best model in terms of each measure.

^b^AUC-ROC: area under the curve of receiver operating characteristic curve.

^c^AUC-PR: area under the curve of precision-recall curve.

^d^MCC: Matthews correlation coefficient.

**Table 9:** Top 10 input variables ranked by Gain (with answering time condition).

Input variable		Gain
**Analysis 1**		
	Nine questions about campus life (number of “yes”)	0.216
	“I have a strong anxiety about my campus life.”	0.088
	“I’m worried about my future.”	0.033
	2nd principal component score of *Diff*_*RT*_^a^	0.031
	2nd principal component score of *Dens*_*RT*_^b^	0.030
	x coordinate of response time stamp	0.026
	Mean of “I had some trouble with someone close to me” in department	0.025
	Individual mean of standardized log RT^c^	0.021
	1st principal component score of *Diff*_*RT*_	0.021
	“I always feel busy with my academic work.”	0.020
**Analysis 2**		
	Nine questions about campus life (number of “yes”)	0.131
	“I have a strong anxiety about my campus life.”	0.080
	y coordinate of response time stamp	0.029
	2nd principal component score of *Diff*_*RT*_	0.028
	Years of university	0.026
	Individual SD of *Dens*_*RT*_	0.024
	1st principal component score of standardized log RT	0.024
	“I’m worried about my future.”	0.023
	Individual SD of standardized log RT	0.023
	Kernel density of *s*	0.023

^a^*Diff_RT_* is the difference between the standardized log RT of each item and individual mean.

^b^*Dens_RT_* is the kernel density of the standardized log RT of each item.

^c^RT: response time.

**Table 10:** Top 10 input variables ranked by Gain (without answering time condition).

Input variable		Gain
**Analysis 1**		
	Nine questions about campus life (number of “yes”)	0.200
	“I have a strong anxiety about my campus life.”	0.089
	Mean of “I had some trouble with someone close to me.” in department	0.035
	sleeping hours	0.032
	“I'm worried about my future.”	0.029
	Faculty and department	0.024
	years of university	0.023
	“I always feel busy with my academic work.”	0.023
	Proportion of the same sex in department	0.023
	Hours of internet use	0.022
**Analysis 2**		
	Nine questions about campus life (number of “yes”)	0.143
	“I have a strong anxiety about my campus life.”	0.119
	“I'm worried about my future.”	0.047
	“I always feel busy with my academic work.”	0.031
	Proportion of the same sex in department	0.031
	Years of university	0.030
	“It takes me a long time to get used to new surroundings.”	0.030
	The academic year of admission	0.028
	Hours of internet use	0.027
	Mean of “I have a strong anxiety about my campus life” in department	0.023

**Table 11:** Top 10 input variables ranked by Shapley additive explanations (SHAP; with answering time condition).

Input variable		SHAP
**Analysis 1**		
	Nine questions about campus life (number of “yes”)	0.027
	“I have a strong anxiety about my campus life.”	0.015
	“I’m worried about my future.”	0.006
	Mean of “I had some trouble with someone close to me.” in department	0.005
	2nd principal component score of *Dens*_*RT*_^*a*^	0.005
	“I always feel busy with my academic work.”	0.005
	2nd principal component score of *Diff*_*RT*_^*b*^	0.005
	Years of university	0.004
	“It takes me a long time to get used to new surroundings.”	0.004
	x coordinate of response time stamp	0.003
**Analysis 2**		
	Nine questions about campus life (number of “yes”)	0.019
	“I have a strong anxiety about my campus life.”	0.016
	Years of university	0.006
	“I’m worried about my future.”	0.006
	The academic year of admission	0.005
	“It takes me a long time to get used to new surroundings.”	0.005
	Have meals (breakfast)	0.005
	“I always feel busy with my academic work.”	0.005
	Have lost over 3 kg in a month (presently)	0.004
	Proportion of the same sex in department	0.004

^a^*Dens_RT_* is the kernel density of the standardized log RT of each item.

^b^*Diff_RT_* is the difference between the standardized log RT of each item and individual mean.

**Table 12:** Top 10 input variables ranked by Shapley additive explanations (SHAP; without answering time condition).

Input variable		SHAP
**Analysis 1**		
	Nine questions about campus life (number of “yes”)	0.028
	“I have a strong anxiety about my campus life.”	0.015
	Mean of “I had some trouble with someone close to me.” in department	0.006
	“I’m worried about my future.”	0.006
	Years of university	0.005
	“I always feel busy with my academic work.”	0.005
	The academic year of admission	0.003
	“It takes me a long time to get used to new surroundings.”	0.003
	Sleeping hours	0.003
	Have lost over 3 kg in a month	0.003
**Analysis 2**		
	“I have a strong anxiety about my campus life.”	0.018
	Nine questions about campus life (number of “yes”)	0.016
	“I’m worried about my future.”	0.008
	The academic year of admission	0.007
	“I always feel busy with my academic work.”	0.007
	Years of university	0.007
	“It takes me a long time to get used to new surroundings.”	0.007
	Have meals (breakfast)	0.006
	Have lost over 3 kg in a month (presently)	0.005
	Age	0.004

**Figure 3:**
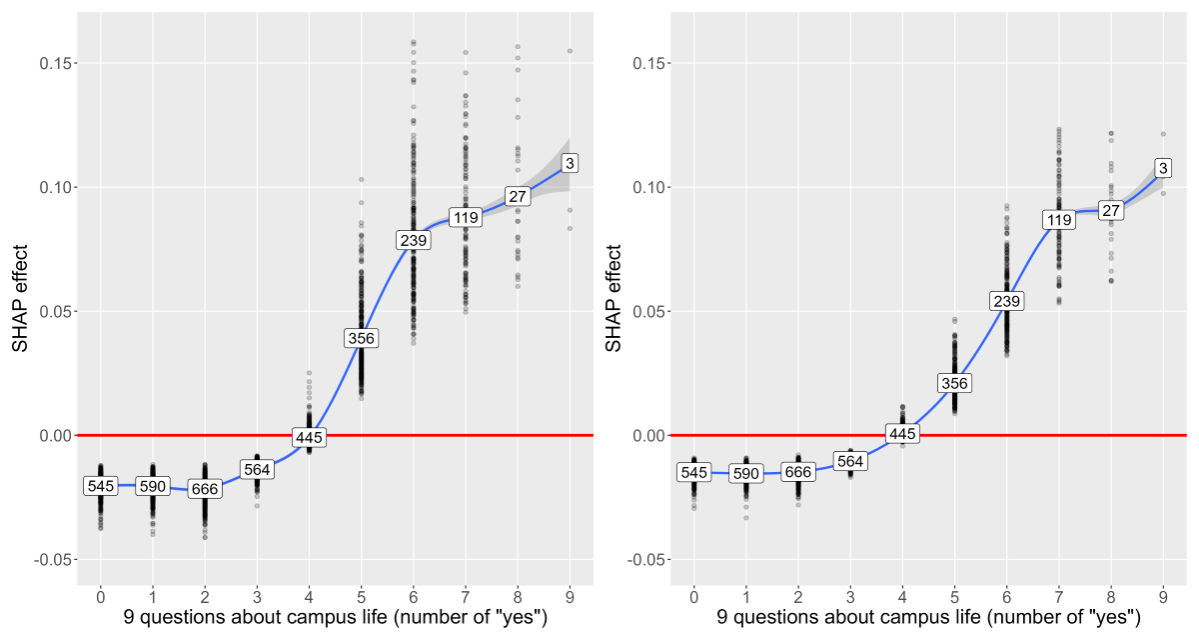
Shapley additive explanations (SHAP) dependence plot of the number of “yes” answers to 9 questions about campus life on “with” condition (left: analysis 1; right: analysis 2).

**Figure 4:**
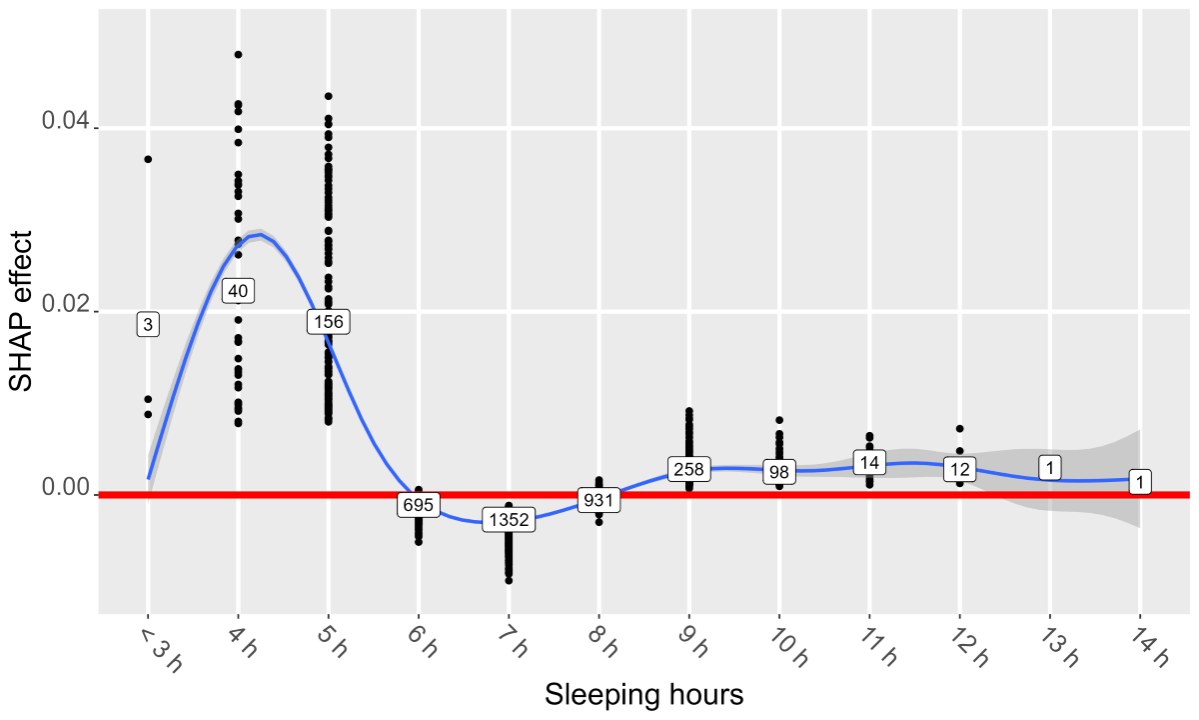
Shapley additive explanations (SHAP) dependence plot of sleeping hours (analysis 1, “without” condition).

**Figure 5:**
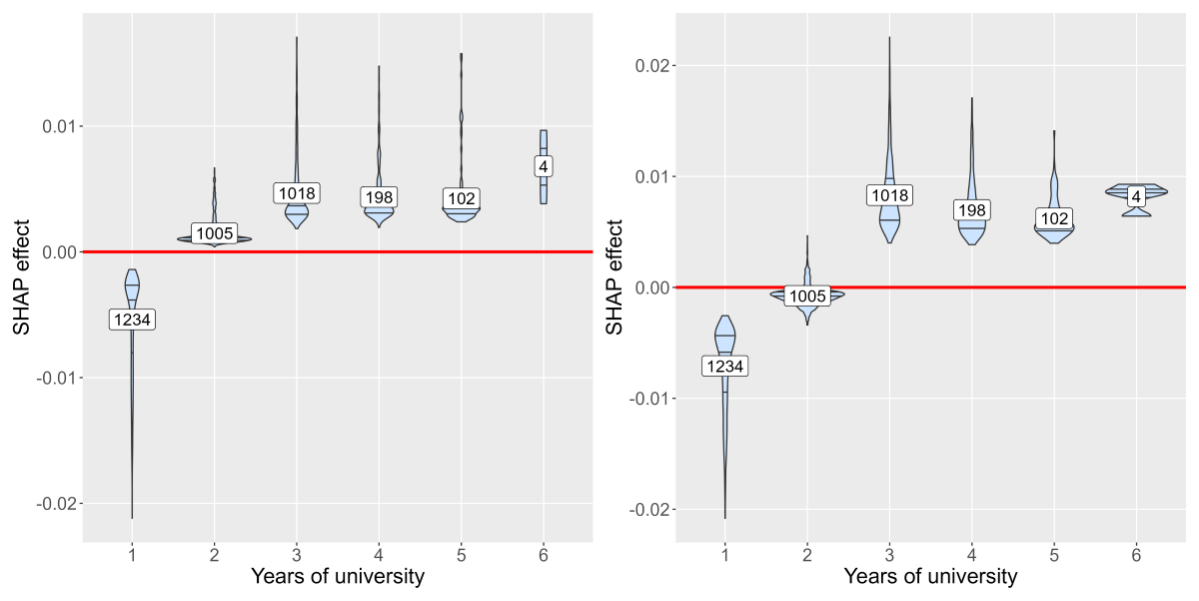
Shapley additive explanations (SHAP) violin plot of years of university on “with” condition (left: analysis 1; right: analysis 2).

**Figure 6:**
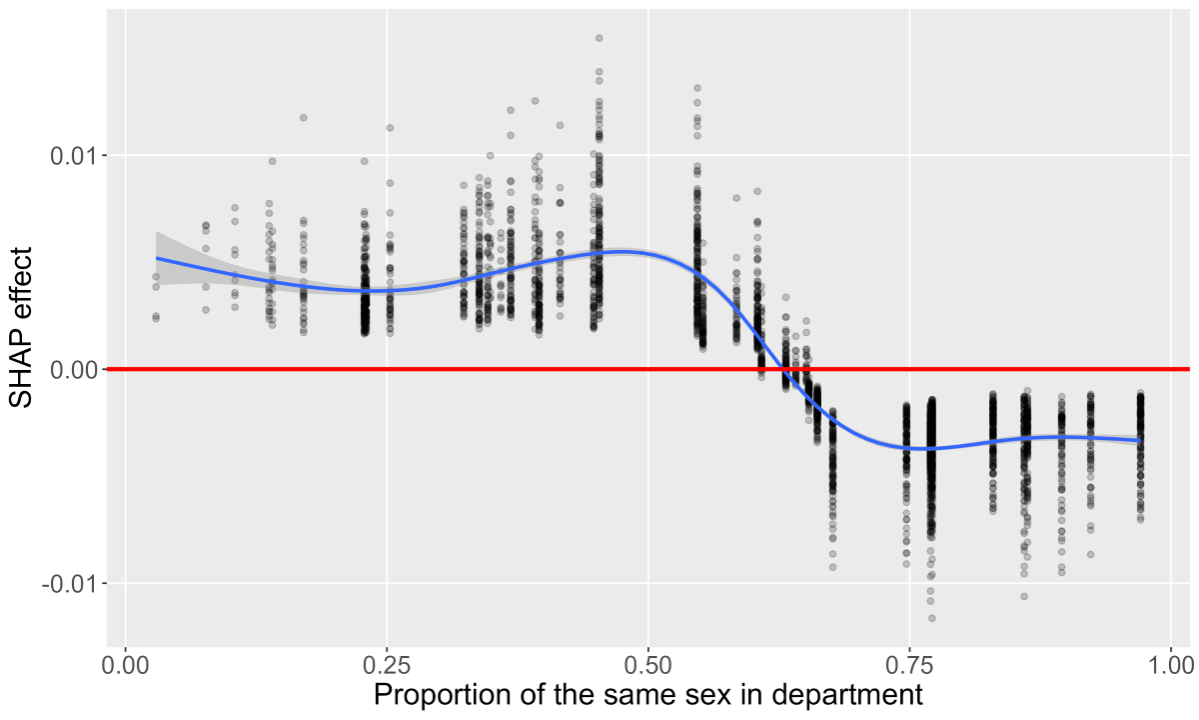
Shapley additive explanations (SHAP) dependence plot of the proportion of the same sex in respondent’s department (analysis 2, “with” condition).

**Figure 7:**
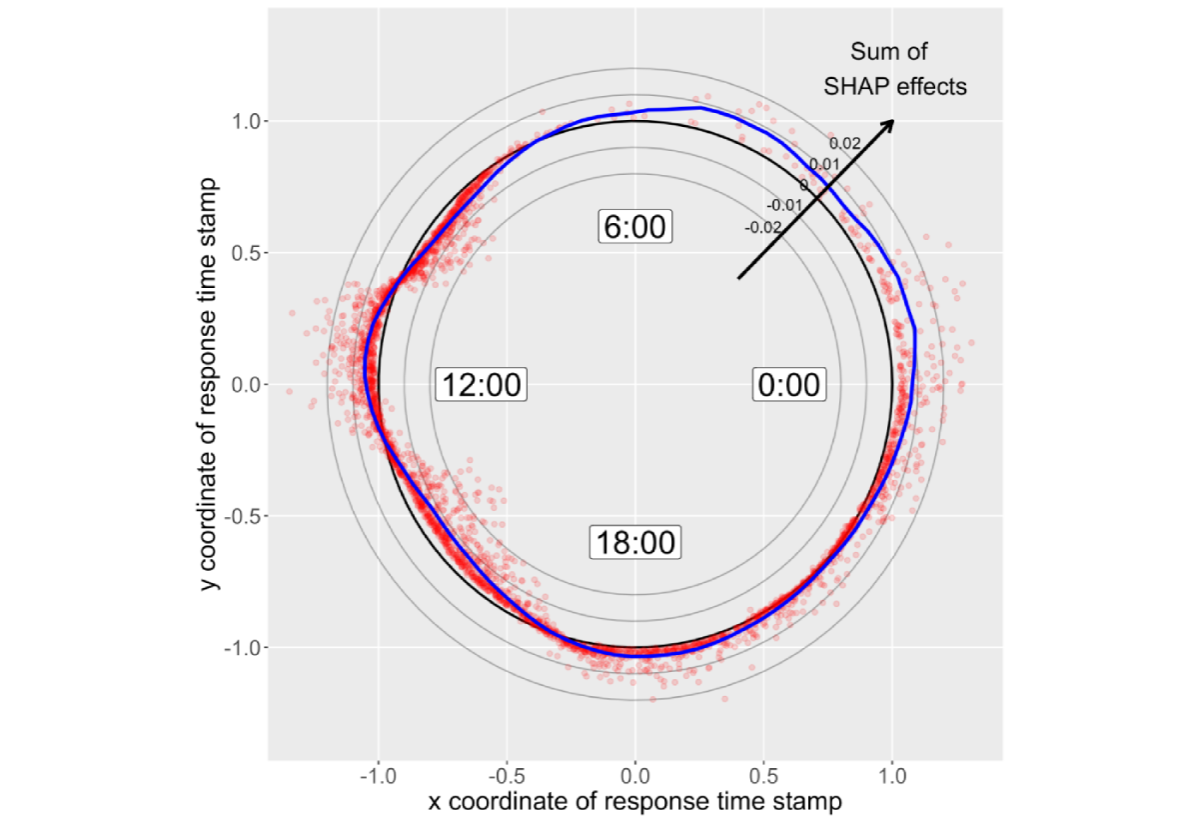
Shapley additive explanations (SHAP) dependence plot of xy coordinates of response time stamp (analysis 1, “with” condition).

**Figure 8:**
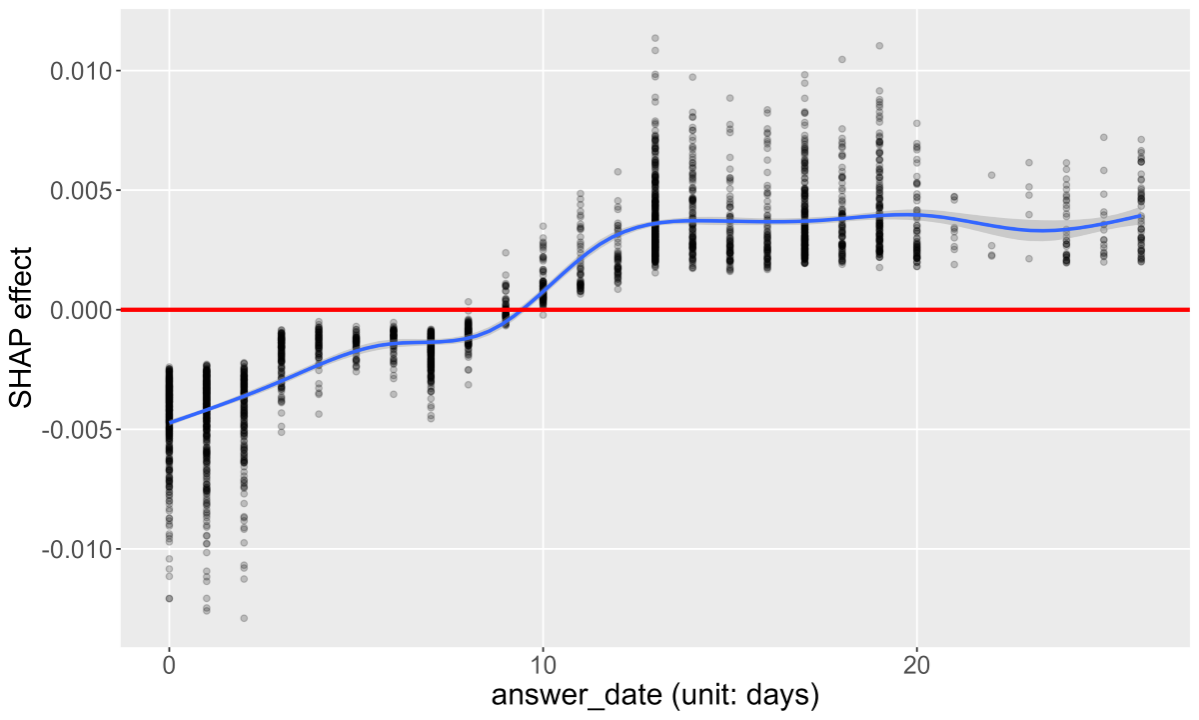
Shapley additive explanations (SHAP) dependence plot of answer date (analysis 2, “with” condition).

Figures 3-8 show SHAP dependence plots or violin plots of some interpretable input variables with high feature importance on average: the number of “yes” answers to *nine questions about campus life*, sleeping hours, years of university, proportion of the same sex in respondent’s department, xy coordinates of RT stamp, and answer date. The blue lines indicate smoothed conditional mean based on the generalized additive model. Figure 3 shows that the averages of the SHAP effect were positive for ≥5 “yes” answers in 9 questions in common to both analyses 1 and 2, indicating that many campus life problems increased predictive probability. Figure 4 shows that the averages of the SHAP effect were positive for <6 hours or >8 hours of sleep, indicating that too short or too long sleep increased predictive probability. Figure 5 shows that the averages of the SHAP effect were positive for third- or higher-year students. Focusing on second-year students, the averages of the SHAP effect were positive in analysis 1 and negative in analysis 2, indicating that predictive probability decreased. Figure 6 shows that the averages of the SHAP effect were positive for fewer proportion of the same sex in the department, indicating high predictive probability. All the top 5 departments with a low proportion of the same sex were in the faculty of science and engineering; female students were 2.8% (4/141) to 14.4% (18/125). The proportion of men in the international department was only 17.1% (32/187).

Figure 7 shows the SHAP dependence plot on xy coordinates of the RT stamp. Both axes represent the realized values of the input. The correspondence between the RT stamp and the coordinates is shown in the graph. For example, if an answer was recorded at 00:00 (midnight), its RT stamp was transformed to (x,y)=(1,0). Red dots were plotted in conjunction with the sum of the SHAP values on the x and x coordinates. Their dots move outside the thick black circle (x^2^ + y^2^ = 1) if the sum is positive. As a result, if a respondent’s answer was recorded at 00:00 (midnight) and the sum of SHAP values was 0.02, a red dot according to the respondent was plotted at (x,y)=(1,0)*0.02*10=(1.2,0). Note that 10 is the magnification ratio determined arbitrarily for visibility. The thin black lines are circles of x^2^+y^2^=0.8,0.9,1.1,1.2, indicating SHAP values of −0.02, −0.01, 0.01, and 0.02. The figure suggests that respondents whose answers were recorded at midnight tended to show large SHAP values, indicating a high predictive probability. By contrast, those whose answers were recorded in the morning (approximately 7:00-11:00) or afternoon (approximately 13:00-17:00) were plotted inside the thick black circle, indicating negative SHAP values. Figure 8 suggests that an ascending relationship exists between the answer date and SHAP. This indicates that students who responded to the survey more than 9 to 10 days after the announcement were more likely to be predicted as having mental health problems.

## Discussion

### Principal Findings

Among all the models, elastic net and LightGBM performed the best in analyses 1 and 2 (Tables 6 and 7). We adopted the LightGBM in this study based on the confusion matrix (Figure 2); however, the elastic net can also have an adequate ability to predict mental health problems not only in real time but also 1 year in the future.

Some of the answering time findings are listed in Table 8. Whether the *with* condition outperformed the *without* condition depended on the performance measure considered. This is possibly because the LightGBM can obtain excellent performance even without answering time–related variables on these data. However, as shown in Tables 9 and 11, certain variables are apparently helpful in improving the prediction and affecting the prediction probability. In summary, it is difficult to determine whether we should use answering time–related variables to predict mental health problems.

Focusing on input variables, those from *nine questions about campus life* improved performance (Tables 9 and 10) and were commonly effective for prediction (Tables 11 and 12). Comparing Gain and SHAP in *with* condition (Tables 9 and 11), it is suggested that some input variables converted from the answering time substantially impacted the learning. With respect to the *without* condition (Tables 10 and 12), it is suggested that the input variable, years of university, could improve the prediction to some extent.

### Detailed Discussion on Input Variables

In Tables 9-12, the input variables from *nine questions about campus life* held a top-ranking position. In particular, the item “I have a strong anxiety about my campus life” had a stable and powerful influence on prediction. The questions were easy to use because there were only 9 yes or no questions. Figure 3 suggests that students were more likely to have mental health problems when they responded positively to ≥5 items of the 9 questions. Furthermore, the variable *nine questions about campus life* could be used by faculty members to advise student orally in addition to health staff members. Their response will be useful for whole-university support and for early detection.

Sleeping hours affected learning and prediction more in analysis 2 than in analysis 1 on the “without” condition (Tables 10 and 11). This implies that sleeping hours were less effective in learning and predicting mental health problems within 1 year. Sleeping hours may be a fluid measure that is affected by lifestyle and busyness. Figure 4 suggested that 6 to 8 hours of sleep were desirable in terms of mental health.

In Japan, 7 hours and 22 minutes are the average sleeping hours [57]. More than one-third of the students in this survey slept for 7 hours. Sleeping too little or too much is a representative symptom of mental health disorder (eg, depressive disorders) [58]. Even without any disorder, sleep diminution because of overwork and busyness could cause mental health problems, and sleeping too much may cause problems in academics. It can be useful for students sleeping <6 or >8 hours to be instructed on how to improve their life rhythm using flyers. Faculty members should keep in mind the need for students to sleep for >6 hours when handing assignments.

Years of university was an effective parameter in both analyses 1 and 2, and in both with and without conditions (Figure 5). The average SHAP effect of second-year students indicated that predictive probability decreased in the second year. It was reported that first- and last-year students tend to experience academic stress [59]. The results depicted in Figure 5 may imply that students adapt to the campus life from the beginning of the second year to their third year. Although some universities hold health surveys only for first-year students, this study indicated that third- or higher-year students should also be monitored.

Figure 6 is consistent with the hypothesis that some students who belonged to departments with few same-sex students may have had difficulties. By contrast, a low proportion of the same sex in a department equals a high proportion of the other sex in that department, indicating a low predictive probability of the other sex. Considering that even an approximately 0.50 proportion of the same sex in the department showed positive averages of the SHAP effect, it is unclear how much the male:female ratio is desirable for students’ mental health. A low proportion of female students in the science and engineering departments reflected the gap in field of science, technology, engineering, and mathematics. The aspect of sex associated with these data was only male or female; the lesbian, gay, bisexual, transgender, queer community should be considered in future studies. Regardless of sex, being in a minority in a department should be a parameter to be examined; such examinations may afford opportunities for interaction.

There are certain findings regarding the input variables generated based on answering time. Concerning the RT stamp, the results depicted in Figure 7 were partly consistent with the hypothesis that RT stamps may reflect students’ life rhythm and sleep quality, which may be related to their mental health state. Students whose answers were recorded at midnight may have stayed up late, which may have been related to mental health problems. In contrast, it is unclear why students whose answers were recorded at noon (approximately 12:00) or evening (approximately 18:00) had negative SHAP values. This might suggest that they had been alone when groups of other students enjoyed lunch or after school. RT stamp data have not been used before; such data can be informative for understanding students’ lifestyles. Although the answer date was not ranked in Tables 9-12, the results depicted in Figure 8 were consistent with the hypothesis that late answer data may have reflected some problems. We believe that the high SHAP of late responses may be caused by the lack of ability to check their emails and obtain essential information from them. Students who answered the survey at a later date should be asked about difficulty in schoolwork (eg, submission of assignments) in health checkup interview. Demonstrating that answering time affects the prediction and learning of mental health problems is meaningful because this parameter can easily be collected through a web survey.

### Limitations

The data were collected from only one university during COVID-19; therefore, the model cannot be generalized to other universities and ages, whereas the approach can be. Campus life during COVID-19 differed from that without the virus. Moreover, this study could not reveal the condition of the 1994 students who did not provide answers in 2020 and 2021, even though they accounted for a major proportion of the population and may have had some mental health problems. The *nine questions about campus life*, which were the most powerful items in this study, are unique to University A and are not widely used. This model should be tested in other time periods or with other universities’ data. Using this model to call for psychological counseling for students at risk in a specific field is entirely different. This model should be evaluated to determine whether it helps in precisely detecting students experiencing mental health problems and to estimate the impact on cost reduction.

### Conclusions

Students’ mental health problems were predicted in real time and for 1 year in the future. The *nine questions about campus life*, especially the question “I have a strong anxiety about my campus life” was an overwhelmingly powerful item. It was indicated that demographic data (eg, years of university, proportion of the same sex in department, etc) and behavioral data (sleeping hours and answering time), as well as self-rating items were effective.

The developed model itself should be adjusted for each university because it depends on the items used in the survey of the cooperating university. Nevertheless, this model demonstrates the possibility of synergistically using the characteristics of health surveys and advantages of ML. Consequently, it can be used for predicting mental health status from existing health data without a mental health scale.

Furthermore, the impact of some items on the prediction was discussed. These findings can improve health survey items and define the criteria for inviting to student counseling. Accurate calls for student counseling will lead to early detection and intervention and operational efficiency.

## Abbreviations

AUC-PR: area under the curve of precision-recall curve
AUC-ROC: area under the curve of receiver operating characteristic curve
GBDT: gradient boosting decision tree
MCC: Matthews correlation coefficient
ML: machine learning
RT: response time
SHAP: Shapley additive explanations
